# Estimation of chronic kidney disease incidence from prevalence and mortality data in American Indians with type 2 diabetes

**DOI:** 10.1371/journal.pone.0171027

**Published:** 2017-02-06

**Authors:** Pavithra Vijayakumar, Annika Hoyer, Robert G. Nelson, Ralph Brinks, Meda E. Pavkov

**Affiliations:** 1 National Institute of Diabetes and Digestive and Kidney Diseases, National Institutes of Health, Phoenix, Arizona, United States of America; 2 Institute for Biometry and Epidemiology, German Diabetes Center Duesseldorf, Germany; 3 Division for Diabetes Translation, Centers for Disease Control and Prevention, Atlanta, Georgia, United States of America; The University of Tokyo, JAPAN

## Abstract

The objective was to estimate chronic kidney disease (CKD) incidence rates from prevalence and mortality data, and compare the estimates with observed (true) incidence rates in a well-characterized population with diabetes. Pima Indians aged 20 years and older with type 2 diabetes were followed from 1982 through 2007. CKD was defined by estimated GFR (eGFR) <60 ml/min/1.72 m^2^ or albumin-to-creatinine ratio (ACR) ≥30 mg/g. True CKD incidence and mortality rates were computed for the whole study period, and prevalence for the intervals 1982–1994 and 1995–2007. Estimated age-sex stratified CKD incidence rates were computed using illness-death models of the observed prevalences, and of the whole-period mortality rate ratio of CKD to non-CKD persons. Among 1201 participants, 616 incident events of CKD occurred during a median follow-up of 5.6 years. Observed CKD prevalence was 56.9% (95%CI 53.7–60.0) and 48.0% (95%CI 45.2–50.8) in women; 54.0% (95%CI 49.9–58.1) and 49.6% (95%CI 46.0–53.3) in men, across the two periods. Mortality rate was 2.5 (95%CI 1.9–3.3) times as high in women with CKD and 1.6 (95%CI 1.3–2.1) times as high in men with CKD, compared to women or men without CKD. In women, estimated CKD incidence increased linearly from 25.6 (95%CI 4.2–53.0) to 128.6 (95%CI 77.1–196.6) with each 5-year age group up to 69 years, and to 99.8 (95%CI 38.7–204.7) at age ≥70. In men, estimated CKD incidence increased form 28.5 (95%CI 3.8–71.2) at age 20–24 years to 118.7 (95%CI 23.6–336.7) at age ≥70. Age-sex-stratified estimated incidence reflected the magnitude and directional trend of the true incidence and were similar to the true incidence rates (p>0.05 for difference) except for age 20–24 in women (p = 0.008) and age 25–29 in men (p = 0.002). In conclusion, the estimated and observed incidence rates of CKD agree well over 25 years of observation in this well characterized population with type 2 diabetes.

## Introduction

Chronic kidney disease (CKD) affects 39.2% of adults with diabetes in the United States [1]. When adjusted for demographic characteristics of the population, the prevalence of CKD in 2007–2012 was slightly but non-significantly lower than in 1988–1994, even though the death rate remained largely stable in the general population and tended to decline among persons with diabetes over the same time period [2]. This observation suggests the incidence of CKD due to diabetes may have declined over this period. Yet, national estimates of CKD incidence are essentially unknown. Although the National Health and Nutrition Examination Survey cycles allow us to estimate national prevalence of CKD and overall mortality rates with reasonable precision, the serial cross-sectional design precludes computation of true incidence rates of CKD. Obtaining true incidence estimates would require regular albuminuria and kidney function testing over a long period of time, which is expensive and logistically difficult to perform in the absence of population registries. It is possible, however, to overcome these challenges by applying the illness-death model–a well-described [3,4] mathematical relationship between incidence, prevalence and mortality–to observational epidemiologic data.

In this study, we use observations from a well-characterized population with type 2 diabetes to estimate CKD incidence rates from observed prevalence and mortality data, and then compare these estimates with true CKD incidence rates in this population. We demonstrate that estimating CKD incidence using the illness-death model represents a reliable method for estimating incidence rates from cross-sectional studies.

## Materials and methods

### Study population and design

A longitudinal study of diabetes and related conditions was conducted in the Gila River Indian Community in southern Arizona from 1965 through 2007 [5]. Participants predominantly of Pima or Tohono O’odham heritage were invited to participate in biennial research examinations. Biochemical tests and an oral glucose tolerance test were administered at these research examinations, and detailed medical and familial histories were recorded. Measurements of urine albumin were conducted in all examinations performed on or after July 1, 1982. For the present study, we selected participants who had type 2 diabetes from this longitudinal population-based study and who were 20 years and older at the time of their first examination after July 1, 1982. Diabetes in the Pima Indians is entirely type 2 diabetes mellitus, including in children and adolescents, and it is characterized by the lack of insulin dependence [6], absent or low levels of islet cell and glutamic acid decarboxylase antibodies [7, 8], and absence of strong linkage or association with maturity-onset diabetes of youth loci [9–11]. All participants were then followed until death or their last research examination before the end of the study period, December 31, 2007, whichever came first. Those with missing serum creatinine measures were excluded from analysis (representing 2.09% of the initial cohort).

Diabetes was diagnosed by a 2-hour post-load plasma glucose level ≥11.1 mmol/L (200 mg/dL). The date of diabetes diagnosis was determined from these research examinations or from review of clinical records if diabetes was diagnosed during routine medical care. Serum creatinine was measured by a modified Jaffé reaction with calibration traceable to an isotope-dilution mass spectrometry measurement procedure [12]. Albumin and creatinine concentrations were measured in urine specimens collected at the end of the glucose-tolerance test. Urinary albumin was measured by nephelometric immunoassay and concentrations below the threshold detected by the assay (6.8 mg/L) were set to this value in the analyses. Elevated albuminuria was defined by an ACR ≥30 mg albumin/g creatinine. The estimated glomerular filtration rate (eGFR) was computed by the CKD-EPI equation [13]. CKD was defined by eGFR <60 ml/min/1.72 m^2^ or ACR ≥30 mg/g.

The study was approved by the Review Board of the National Institute of Diabetes and Digestive and Kidney Diseases. Each participant gave written informed consent.

### Statistical analysis

#### Observed (true) CKD incidence rate, prevalence, and mortality

Observed incidence of CKD was calculated for the whole study period as the number of new CKD events per 1,000 person-years at risk. Follow-up extended from the date of the first diabetic research examination after the age of 20 years without CKD to first date with CKD or, in those who did not develop CKD, to the date of the last research examination within the study period. The age- and sex-specific remission rates *r* were computed among those with pre-dialysis CKD as the count of transitions to the "non-CKD" state (numerator) divided by the person-time at risk for remission (denominator); only the first remission event was counted. Observed prevalence of CKD was computed for the calendar intervals 1982–1994 and 1995–2007 using data from the research examination closest to the midpoint of each period. Observed death rates were computed for the whole study period as the number of subjects without or with CKD who died of any cause per 1,000 person-years of follow-up. The period at risk for the without CKD death rate began at the first diabetic examination and ended at the date of death, onset of CKD, or December 31, 2007, whichever came first. The period at risk for those with CKD death rate began at the first diabetic examination with CKD and ended at the date of death or December 31, 2007, whichever came first.

#### Estimated CKD incidence rate

**Fig 1:**
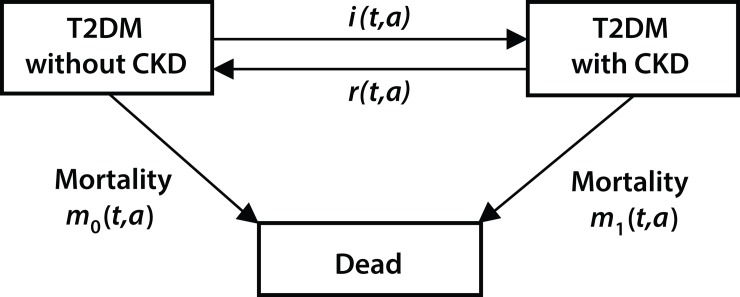
Illness-death model with transition rates (*i*, *r*, *m*_*0*_, *m*_*1*_). CKD, chronic kidney disease; T2DM, type 2 diabetes mellitus.

To estimate incidence of CKD we used the illness-death model shown in Fig 1 [14]. Living persons with diabetes aged *a* at calendar time *t* are either without CKD or with CKD. Persons may change status according to the transition rates *i*, *r*, *m*_0_, *m*_1_, where *i* represents CKD incidence, *r* is the CKD remission rate, *m*_*0*_ the death rate among persons with diabetes without CKD, and *m*_*1*_ the death rate among persons with both diabetes and CKD.

Equation (3) in Brinks and Landwehr [14] solved for the incidence rate *i* leads to the following equation:

$$i=\frac{\partial p+rp}{1-p}+p(m_{1}-m_{0})$$ (1)

Where ∂*p* represents change of prevalence in time (partial derivative), *rp* is remission rate, *m*_*0*_ death rate without CKD, *m*_*1*_ the death rate with CKD, and *p* is the prevalence in the middle (t*), between the first point in time (t1) and the second (t2), i.e.

$$t^{*}=0.5\times (t_{1}+t_{2})$$

*p*(t*, *a*) is estimated from the two surveyed prevalences:

$$p(t^{*},a)=\frac{h_{2}}{h_{1}+h_{2}}p(t_{1},a-h_{1})+\frac{h_{1}}{h_{1}+h_{2}}p_{k}(t_{2},a+h_{2})$$

$$h_{1}=t^{*}-t_{1}$$

$$h_{2}=t_{2}-t^{*}$$

*a* = age [15].

We then compared the incidence rate estimated via Eq (1) with the observed incidence rate. The 95% confidence intervals (CIs) for the estimated incidence rates were obtained by a Monte-Carlo simulation (1000 replications). For more details about the method see Brinks and Landwehr [14]. Pointwise differences between the true and estimated incidence values were evaluated by computing the 95% CI of the difference between the true and estimated incidence. If the calculated interval includes zero, there is no evidence for a difference between the true and estimated incidence rates.

Baseline clinical and demographic features of the study population are presented as median and interquartile range (IQR). Body mass index (BMI) was defined as weight divided by the square of height (kg/m^2^). Mean arterial pressure (MAP) was calculated as 2/3 diastolic arterial pressure + 1/3 systolic arterial pressure. A χ^2^ tests for general association were used to examine relationships between time period, hypoglycemic and antihypertensive treatment; median values were compared by the Kruskal–Wallis test.

## Results

Characteristics of the study population listed in Table 1 are presented for the two time periods for which CKD prevalence was computed. In the second period, the study population was younger, with higher BMI and eGFR, and lower fasting glucose levels, ACR, and MAP. Usage of anti-hypertensive and hypoglycemic treatments improved over time.

**Table 1:** Characteristics of the study population with type 2 diabetes for the prevalence periods.

	Prevalence Period		
	1982–1994	1995–2007	p
n (Male/Female)	1502 (565/937)	1957 (711/1246)	
Age (years)	46 (36–57)	44 (36–54)	0.001
Diabetes duration (years)	6.6 (0–14.9)	6.5 (0.7–14.5)	0.35
A1c (%)	8.5 (6.3–10.5)	8.0 (6.4–10.3)	0.36
Fasting glucose (mg/dL)	194 (129–258)	154 (117–230)	<0.001
BMI (kg/m^2^)	32.6 (28.2–38.1)	35.3 (30.8–41.4)	<0.001
MAP (mmHg)	94.0 (86.0–103.3)	91.3 (83.3–100.0)	<0.001
eGFR (ml/min/1.73 m^2^)	110 (97–122)	113 (100–123)	<0.001
ACR (mg/g)	29.3 (13.1–155.9)	23.5 (10.3–98.2)	<0.001
Hypoglycemic treatment (%)	42	50	<0.001
Anti-hypertensive treatment (%)	21	46	<0.001

Values are shown as median (interquartile range). A1c, haemoglobin A1c; ACR, albumin/creatinine ratio; BMI, body mass index; eGFR, estimated glomerular filtration rate; MAP, mean arterial pressure.

Among 1201 participants (783 women, 418 men) with type 2 diabetes and without CKD at baseline, 616 (407 women, 209 men) incident events of CKD occurred during a median of 5.6 years of follow-up (IQR 2.9 to 9.9 years); 19 of these represented incident dialysis cases. The observed age- and sex-stratified incidence rates of CKD are shown in Table 2. Overall, observed CKD incidence was 76.5 events/1,000 person-years (95% CI 70.6–82.8), and was similar in women (74.9 events/1,000 person-years, 95% CI 67.8–82.6) and men (79.7 events/1,000 person-years, 95% CI 69.3–91.3).

**Table 2:** Age- and sex-specific observed (true) and estimated incidence rate of chronic kidney disease in the cohort with type 2 diabetes during the study period (1982–2007). CI, confidence interval.

Age (years)	Number of Events	Person-years	True Incidence (events/1,000 person-years)	95% CI	Estimated Incidence (events/1,000 person-years)	95% CI	95% CI for Difference	P for Difference
	**Women**							
20–24	21	231.22	90.82	56.22–138.83	25.61	4.28–53.06	**17.23–113.17**	**0.008**
25–29	17	458.05	37.11	21.62–59.42	35.37	20.31–51.19	-22.66–26.14	0.89
30–34	47	686.33	68.48	50.32–91.06	51.09	35.88–66.07	-7.95–42.74	0.17
35–39	51	812.31	62.78	46.75–82.55	61.64	45.95–77.19	-22.62–24.89	0.93
40–44	51	840.45	60.68	45.18–79.78	68.32	51.29–85.10	-31.83–16.54	0.72
45–49	59	781.63	75.48	57.46–97.37	80.93	61.63–101.52	-33.66–22.76	0.85
50–54	51	598.71	85.18	63.42–112.0	87.60	67.39–109.17	-34.46–29.61	0.94
55–59	39	436.87	89.27	63.48–122.04	100.55	75.07–132.82	-52.40–29.84	0.76
60–64	32	304.21	105.19	71.95–148.5	113.62	77.03–151.42	-61.80–44.94	0.88
65–69	26	172.23	150.96	98.61–221.19	128.66	77.19–196.69	-63.29–107.89	0.62
70-UP	13	109.13	119.13	63.43–203.71	99.89	38.71–204.77	-89.45–127.93	0.74
			**Men**					
20–24	11	131.8	83.46	41.66–149.33	28.58	3.85–71.20	-8.61–118.38	0.09
25–29	23	167.47	137.33	87.06–206.07	38.48	18.36–59.03	**35.96–61.73**	**0.002**
30–34	11	265	41.51	20.72–74.27	62.10	39.74–85.29	-55.74–14.55	0.46
35–39	29	406.63	71.32	47.76–102.42	76.52	51.10–102.19	-42.61–32.21	0.92
40–44	33	454.04	72.68	50.03–102.07	65.54	41.73–88.74	-27.92–42.20	0.70
45–49	30	372.29	80.58	54.37–115.04	54.38	30.56–77.124	-12.03–64.44	0.18
50–54	31	308.37	100.53	68.30–142.69	60.69	33.76–89.25	-6.57–86.23	0.09
55–59	14	250.22	55.95	30.59–93.87	72.03	36.74–116.68	-67.05–34.89	0.76
60–64	14	140.25	99.82	54.57–167.48	82.22	31.01–146.67	-63.22–98.41	0.68
65–69	10	77.53	128.98	61.85–237.20	116.12	37.70–237.42	-120.02–145.74	0.85
70-UP	3	49.18	61.00	12.58–178.28	118.79	23.66–336.79	-234.92–119.34	0.74

Among those with pre-dialysis CKD, 341 (249 women, 92 men) experienced remission of CKD during a median follow-up of 4.8 years (IQR 2.3 to 9.6 years); all except three of these remissions were due to reduction of ACR from ≥30 mg/g to <30 mg/g. The overall CKD remission rate was 50.2 events/1,000 person-years (95% CI 45.0–55.8); CKD remission rate was 52.9 events/1,000 person-years in women (95% CI 46.6–60.0), and 44.0 events/1,000 person-years in men (95% CI 35.5–54.0) (S1 Table). In the present study, when defining CKD by an eGFR <60 ml/min/1.73 m^2^, the overall incidence of CKD was 25.4 events/1,000 person-years (95% CI 21.3–30.1) in men and 21.9 events/1,000 person-years (95% CI 19.3–24.8) in women.

Observed CKD prevalence estimates for the first and second periods are shown in S2 Table, stratified by age and sex. Overall, observed prevalence declined from 55.7% (95% CI 53.3–58.3) in 1982–1994 to 48.6% (95% CI 46.4–50.8) in 1995–2007 (p <0.001). In women, CKD prevalence declined from 56.9% (95% CI 53.7–60.0) to 48.0% (95% CI 45.2–50.8) (p <0.001). By contrast, the CKD prevalence remained stable in men at 54.0% (95% CI 49.9–58.1) and 49.6% (95% CI 46.0–53.3) (p = 0.12).

The observed age- and sex-stratified death rates, overall and by presence of CKD, are shown in S3 Table. Observed death rate was 2.5 (95% CI 1.9–3.3) times as high in women with CKD and 1.6 (95% CI 1.3–2.1) times as high in men with CKD, compared to women or men without CKD.

**Fig 2:**
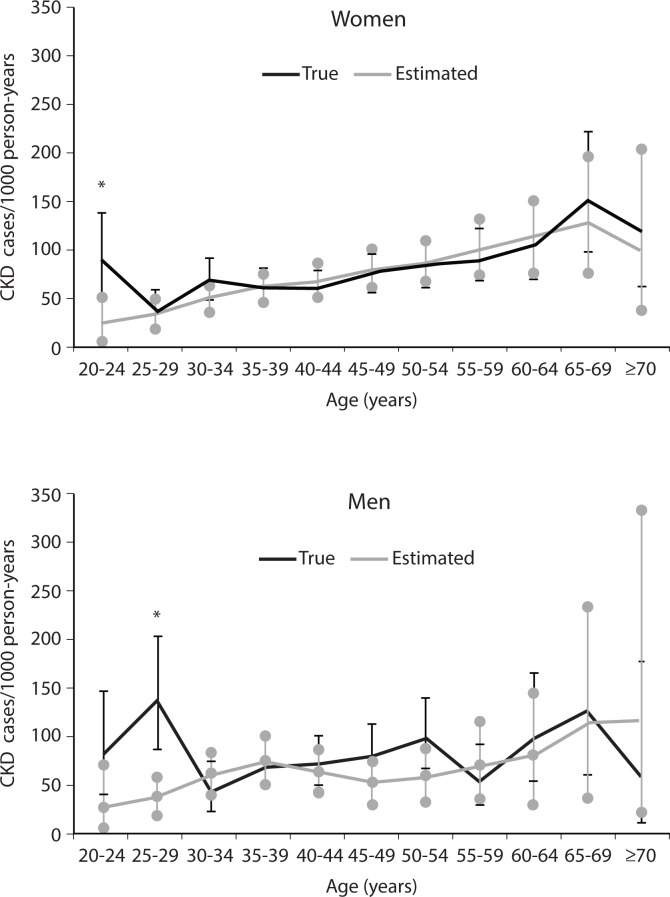
Age- stratified observed and estimated incidence rates of CKD in women (above) and men (below) during the period 1982–2007. The asterisk (*) indicates significant difference between true and estimated CKD incidence.

Age-sex stratified observed (i.e., true) and estimated incidence rates are shown in Fig 2 and Table 2. The magnitude and directional trend of the true incidence with increasing age is reflected in the estimated incidence. The agreement between the true and estimated incidence is very close (p >0.05) in women of all age groups except 20–24 years (p = 0.008). The agreement in men also appears similar, except for age group 25–29 years (p = 0.002) (Table 2).

## Discussion

The estimated incidence rate of CKD based on the illness-death model agreed well with the observed incidence of CKD in this well characterized population with type 2 diabetes. Estimated incidence rates, computed from observed prevalence and mortality data, reproduced the true incidence rates particularly well in women, largely due to a greater number of women than men in each 5-year age group.

Why is it important to know incidence rate of CKD? Although the term ‘epidemic’ has been used in recent years to describe the increasing prevalence of CKD, a true epidemic would require an increasing incidence of the disease. Unlike prevalence, CKD incidence rate is specifically reflecting exposure to particular risk factors and therefore its measure helps characterize factors contributing to disease onset and progression, estimate how changes in these exposures impact development of disease over time, and evaluate the effectiveness of prevention strategies and health interventions. Knowing how much of the prevalence is due to incidence of a disease over time has therefore important implications for the allocation of limited public and healthcare resources for the prevention and treatment of kidney diseases.

Except for reports on the incidence of end-stage renal disease [16, 17], true incidence (i.e., incidence density rate) of diabetic CKD is seldom available in population based studies, in part because this measure of disease occurrence requires a cohort large enough or with considerable follow-up time to analyze the progression to CKD among people at various levels of risk. Furthermore, systematic information on both albuminuria and eGFR is needed to define incident events, and even when prevalence data are available, mortality in both those with and without the disease is necessary to compute CKD incidence rate.

A study among 14,873 participants in the community-based, multicenter, biracial Atherosclerosis Risk in Communities Study (ARIC), used different case definitions to estimate CKD incidence during nine years of follow-up [18]. Because the study had no information on baseline albuminuria, incident events largely reflected risk of advanced kidney disease, defined by increase in serum creatinine, decrease in eGFR, International Classification of Diseases codes for hospitalizations or mortality involving a kidney diagnosis. Among those with diabetes, CKD incidence varied between 18.9 events /1,000 person-years (95% CI 16.4–21.8) and 6.3 events/1,000 person-years (95% CI 5.1–7.7), contingent on case definition. In a prospective cohort study of 3,443 outpatients with type 2 diabetes, sampled from 56 primary health care centers in Spain, the incidence density of stages 3–5 CKD, defined by an eGFR <60 ml/min/1.73 m^2^, was 24.8 events per 1000 person-years (95% CI 21.9–27.9) between 2007–2012 [19]. The incidence rate was found to be lower among patients with less than 10 years duration of diabetes (20.3 events per 1000 person-years, 95% CI 17.3–23.8) than in those with longer duration of diabetes (35.4 events per 1000 person-years, 95% CI 29.2–42.5). In a community-based cohort of 3,313 Iranian adults, the incidence rate of eGFR <60 ml/min/1.73 m^2^ was 21.5 (95% CI 19.9–23.1) per 1,000 person-years during a mean follow-up of 9.9 years; incidence rates among women and men were 28.5 (95% CI 26.2–31.1) and 13.3 (95% CI 11.6–15.2), respectively [20]. The cohort, however, included a small proportion of people with diabetes—about 2%.

Accounting for the remission rate of CKD is important when estimating CKD incidence by the illness-death model. A substantial proportion of persons with moderate albuminuria regress to normoalbuminuria, either spontaneously or as a result of treatment, indicating a measure of reversibility of initial kidney injury rather than inevitable progression to ESRD. Generally, the proportion of persons with type 2 diabetes who regress from moderate to normoalbuminuria is 30%-54%, while the frequency of progression to overt proteinuria is 12%-36% [21–23]. In a previous study among Pima Indians with type 2 diabetes we found that 23% experienced progression and 8% experienced regression of ACR between two consecutive measurements taken within a 6-year period [23]. In the present study the remission rate of CKD was almost entirely due to ACR remission to normal values and to a lesser extent to eGFR surge above 60 ml/min/1.73 m^2^. Thus, accounting for the remission rate in the differential equations will substantially improve the incidence model when all stages of CKD are considered, but may not have any impact on the modelling when CKD is defined by GFR <60 ml/min/1.73 m^2^ only.

The applicability of the illness-death model for extracting incidence density estimates from prevalence and mortality data has been demonstrated previously for chronic diseases such as renal replacement therapy [24], dementia [25], and diabetes [26]. The present study adds to previous information by replicating the model in a non-Caucasian population and accounting for the remission rate of a chronic disease.

The strength of the study derives from including a population in whom type 2 diabetes and CKD are known with greater accuracy than in other studies because of systematic periodic testing as part of the longitudinal follow-up. The study limitations relate to 1) the inclusion of a homogenous cohort of Pima Indians, however, these results are intended to add to previous ones conducted in predominantly Caucasian populations; 2) it is the first time that the method is applied to such a long period, covering 25 years of data. Many changes might have happened during that time, including changes in risk factors, in treatment and mortality, all of which might not be captured in two cross-sections. Even so, the magnitudes of the estimated and observed incidences are similar. Another important limitation is our method of determining if the true and estimated incidences are the same. We tested the null hypothesis that the true and estimated incidences are the same. In effect, this says that they will be declared the same unless the data provide strong evidence that they are different. This could be addressed in some future study by the use of equivalence testing, instead of difference testing.

In conclusion, over 25 years of observation, the estimated incidence rate of CKD based on the illness-death model agreed well with the observed (true) incidence of CKD in Pima Indians with type 2 diabetes. Although we used longitudinal data to compute CKD remission rates, persistence and/or regression of albuminuria can be easily obtained in subsets of cross sectional populations with repeated ACR and eGFR measurements, as has been done for the NHANES population [27]. The present study adds to the body of evidence indicating that the current incidence model is rigorous across populations and chronic diseases outcomes and may be used to estimate incidence density of CKD when longitudinal follow-up is not feasible, but serial cross-sectional data are available.

## Supporting information

S1 Table Observed remission rate of chronic kidney disease by sex and age in type 2 diabetes. (DOCX)

S2 Table Age- and sex-specific observed prevalence of chronic kidney disease in the two time periods of the study. (DOCX)

S3 Table Age- and sex-specific death rates by presence of chronic kidney disease, and the relative mortality of those with compared to those without chronic kidney disease. (DOCX)
