# Supplementary material for: Estimation of chronic kidney disease incidence from prevalence and mortality data in American Indians with type 2 diabetes
Source: PLoS One. 2017 Feb 6;12(2):e0171027. doi: 10.1371/journal.pone.0171027 (PMC5293194; doi:10.1371/journal.pone.0171027)
Supplement: S1 Table — (DOCX) [file pone.0171027.s001.docx]

**S1 Table**. **Observed remission rate of chronic kidney disease by sex and age in type 2 diabetes.**

|  | **All** | | **Women** | | **Men** | |
| --- | --- | --- | --- | --- | --- | --- |
| **Age (years)** | **Remission**  **(events/1,000 person-years)** | **95% CI** | **Remission**  **(events/1,000 person-years)** | **95% CI** | **Remission**  **(events/1,000 person-years)** | **95% CI** |
| 20-24 | 49.95 | 22.84-94.82 | 52.94 | 21.29-109.08 | 41.69 | 5.05-150.6 |
| 25-29 | 46.41 | 27.94-72.48 | 52.94 | 28.94-88.82 | 34.51 | 11.21-80.54 |
| 30-34 | 73.29 | 53.46-98.07 | 73.65 | 49.69-105.14 | 72.58 | 40.62-119.7 |
| 35-39 | 73.21 | 54.51-96.26 | 70.09 | 48.54-97.94 | 80.38 | 46.83-128.7 |
| 40-44 | 52.57 | 38.05-70.81 | 56.6 | 38.46-80.35 | 44.4 | 22.94-77.56 |
| 45-49 | 54.73 | 40.62-72.16 | 69.82 | 49.88-95.07 | 29.36 | 14.08-54 |
| 50-54 | 42.71 | 30.65-57.94 | 47.74 | 32.21-68.16 | 33.18 | 16.56-59.36 |
| 55-59 | 49.23 | 34.84-67.57 | 52.6 | 34.95-76.02 | 41.74 | 20.01-76.76 |
| 60-64 | 31.46 | 19.22-48.59 | 34.91 | 20.34-55.9 | 20.17 | 4.16-58.95 |
| 65-69 | 44.05 | 26.91-68.03 | 40.84 | 22.86-67.35 | 57.65 | 18.72-134.54 |
| ≥70 | 14.81 | 4.81-34.55 | 10.86 | 2.24-31.75 | 32.49 | 3.93-117.35 |
| All | 50.22 | 45.03-55.84 | 52.97 | 46.59-59.97 | 44.02 | 35.49-53.98 |
