# Supplementary material for: Estimation of chronic kidney disease incidence from prevalence and mortality data in American Indians with type 2 diabetes
Source: PLoS One. 2017 Feb 6;12(2):e0171027. doi: 10.1371/journal.pone.0171027 (PMC5293194; doi:10.1371/journal.pone.0171027)
Supplement: S2 Table — (DOCX) [file pone.0171027.s002.docx]

**S2 Table**. **Age- and sex-specific observed prevalence of chronic kidney disease in the two time periods of the study.**

|  | **1982-1994** | | **1995-2007** | |  |
| --- | --- | --- | --- | --- | --- |
| **Age (years)** | **Prevalence (%)** | **95% CI** | **Prevalence (%)** | **95% CI** |  |
| **Women** | | | | | |
| 20-24 | 48.39 | 29.75-67.02 | 33.33 | 21.05-45.61 |  |
| 25-29 | 48.81 | 37.9-59.72 | 21.62 | 12.02-31.22 |  |
| 30-34 | 42.99 | 33.46-52.52 | 46.81 | 38.47-55.15 |  |
| 35-39 | 40.68 | 31.68-49.67 | 36.91 | 29.53-44.28 |  |
| 40-44 | 48.91 | 38.5-59.32 | 46.91 | 39.82-53.99 |  |
| 45-49 | 55.24 | 45.57-64.91 | 47.85 | 40.1-55.6 |  |
| 50-54 | 64.36 | 54.85-73.86 | 51.68 | 43.56-59.8 |  |
| 55-59 | 68.22 | 59.26-77.19 | 51.77 | 40.92-62.61 |  |
| 60-64 | 69.51 | 59.34-79.69 | 62.07 | 51.67-72.47 |  |
| 65-69 | 81.03 | 70.64-91.43 | 68.12 | 56.84-79.39 |  |
| ≥70 | 73.08 | 60.61-85.55 | 76.79 | 65.38-88.2 |  |
| Total | 56.9 | 53.7-60.0 | 48 | 45.2-50.8 |  |
| Men | | | | |  |
| 20-24 | 47.62 | 24.32-70.91 | 15.15 | 2.24-28.06 |  |
| 25-29 | 40.00 | 24.13-55.87 | 49.06 | 35.15-62.97 |  |
| 30-34 | 38.98 | 26.16-51.8 | 48.89 | 38.36-59.42 |  |
| 35-39 | 51.47 | 39.28-63.66 | 42.98 | 33.76-52.21 |  |
| 40-44 | 51.39 | 39.56-63.22 | 43.69 | 33.95-53.43 |  |
| 45-49 | 61.46 | 51.55-71.37 | 52.69 | 42.35-63.03 |  |
| 50-54 | 58.21 | 46.09-70.33 | 60.23 | 49.8-70.66 |  |
| 55-59 | 50.91 | 37.27-64.55 | 58.18 | 44.72-71.64 |  |
| 60-64 | 67.44 | 52.85-82.03 | 60.00 | 44.13-75.87 |  |
| 65-69 | 64.00 | 43.78-84.22 | 57.69 | 37.34-78.04 |  |
| ≥70 | 68.42 | 45.4-91.44 | 68.75 | 43.24-94.26 |  |
| Total | 54 | 49.9-58.1 | 49.6 | 46.0-53.3 |  |
