# Supplementary material for: Estimation of chronic kidney disease incidence from prevalence and mortality data in American Indians with type 2 diabetes
Source: PLoS One. 2017 Feb 6;12(2):e0171027. doi: 10.1371/journal.pone.0171027 (PMC5293194; doi:10.1371/journal.pone.0171027)
Supplement: S3 Table — (DOCX) [file pone.0171027.s003.docx]

**S3 Table**. **Age- and sex-specific death rates by presence of chronic kidney disease, and the relative mortality of those with compared to those without chronic kidney disease.**

|  | **No CKD** | | **CKD** | |  |
| --- | --- | --- | --- | --- | --- |
| **Age (years)** | **Death Rate**  **(events/1,000 person-years)** | **95% CI** | **Death Rate**  **(events/1,000 person-years)** | **95% CI** | **Relative Mortality** |
| **Women** | | | | | |
| 20-24 | 6.11 | 0.74-22.08 | 0 |  | 0 |
| 25-29 | 3.17 | 0.38-11.47 | 2.4 | 0.06-13.4 | 0.75 |
| 30-34 | 1.99 | 0.24-7.17 | 7.55 | 2.45-17.61 | 3.79 |
| 35-39 | 1.45 | 0.18-5.22 | 10.34 | 4.73-19.63 | 7.13 |
| 40-44 | 4.59 | 1.84-9.45 | 9.45 | 4.53-17.39 | 2.05 |
| 45-49 | 7.27 | 3.63-13.01 | 11.09 | 5.91-18.97 | 1.52 |
| 50-54 | 5.86 | 2.36-12.07 | 21.44 | 14-31.41 | 3.65 |
| 55-59 | 6.35 | 2.33-13.82 | 32.22 | 22.8-44.23 | 5.07 |
| 60-64 | 7.17 | 2.33-16.74 | 38.13 | 27.48-51.53 | 5.31 |
| 65-69 | 26.97 | 14.36-46.12 | 44.84 | 32.45-60.41 | 1.66 |
| ≥70 | 46.77 | 30.55-68.54 | 82.73 | 68.3-99.3 | 1.76 |
| Total | 8.09 | 6.44-10.03 | 29.51 | 26.28-33.03 | 3.64 |
| **Men** | | | | | |
| 20-24 | 10.27 | 1.24-37.09 | 0 |  | 0 |
| 25-29 | 0 |  | 8.18 | 0.99-29.56 | - |
| 30-34 | 16.66 | 7.19-32.82 | 12.6 | 4.09-29.4 | 0.75 |
| 35-39 | 4.19 | 0.86-12.26 | 6.95 | 1.89-17.79 | 1.65 |
| 40-44 | 8.25 | 3.32-17 | 17.04 | 9.07-29.14 | 2.06 |
| 45-49 | 15.24 | 7.88-26.63 | 19.35 | 11.27-30.99 | 1.26 |
| 50-54 | 23.2 | 13.26-37.68 | 48.39 | 35.02-65.19 | 2.08 |
| 55-59 | 33.88 | 20.7-52.33 | 35.51 | 23.2-52.03 | 1.04 |
| 60-64 | 17.33 | 6.97-35.71 | 68.38 | 48.62-93.48 | 3.94 |
| 65-69 | 59.56 | 34.7-95.37 | 55.85 | 34.12-86.26 | 0.93 |
| ≥70 | 43.55 | 23.81-73.07 | 128.95 | 95.08-170.97 | 2.96 |
| Total | 18.94 | 15.51-22.91 | 36.76 | 32.03-41.99 | 1.94 |
